# GENETIC DETERMINANTS OF ANTIBIOTIC RESISTANCE AND VIRULENCE IN *Staphylococcus aureus* ISOLATES FROM HOSPITAL SURFACES IN HIGH-RISK AREAS OF LOJA, ECUADOR

**DOI:** 10.21010/Ajidv19i2S.10

**Published:** 2025-10-17

**Authors:** LIMA- QUIZHPE Cristina, ANDRADE- TACURI Carlos, ORTIZ-TEJEDOR Jonnathan, ORELLANA-BRAVO Paola, TOLEDO-ANDRADE Karla

**Affiliations:** * Universidad Católica de Cuenca. Cuenca-Ecuador. https://orcid.org/0000-0003-2599-0728; ** Universidad Católica de Cuenca. Unidad Académica de Salud y Bienestar. Carrera de Odontología. Laboratorio de Biología Molecular y Genética del Centro de Investigación, Innovación y Transferencia de Tecnología de la Universidad Católica de Cuenca (CIITT). Grupo de investigación en Genética y Biología Molecular de Microorganismos (GI-GyBM2). Cuenca, Ecuador. https://orcid.org/0000- 0003-3983-1314; *** Universidad Católica de Cuenca. Unidad Académica de Posgrado. Unidad Académica de Salud y Bienestar. Carrera de Bioquímica y Farmacia. Grupo de investigación en Genética y Biología Molecular de Microorganismos (GI-GyBM2). Cuenca. Ecuador. https://orcid.org/0000-0001-6770-2144.; **** Universidad Católica de Cuenca. Unidad Académica de Salud y Bienestar. Carrera de Odontología. Laboratorio de Biotecnología del Centro de Investigación, Innovación y Transferencia de Tecnología de la Universidad Católica de Cuenca (CIITT). Grupo de investigación en Genética y Biología Molecular de Microorganismos (GI-GyBM2). Cuenca, Ecuador. https://orcid.org/0000-0001-6276-0521.; ***** Odontóloga por la Universidad Católica de Cuenca. Cuenca-Ecuador. https://orcid.org/0000-0002-7559-0645

**Keywords:** *Staphylococcus aureus*, antibiotic susceptibility, virulence factors, Polymerase Chain Reaction

## Abstract

**Background:**

*Staphylococcus aureus* is a microorganism associated with nosocomial infections, characterized by its high pathogenicity and antibiotic resistance, posing a critical risk in hospital environments. This study aimed to determine its presence, antibiotic susceptibility, and the detection of virulence, adhesion, and regulatory genes on hospital surfaces using phenotypic and molecular methods.

**Materials and Methods:**

A total of 200 surface samples were collected from a secondary-level hospital, including clinical wards, ICU, and emergency areas. *S. aureus* was isolated using phenotypic techniques (mannitol, coagulase, DNase) and genotypic methods (detection of *nucA* and *femB*). Antimicrobial susceptibility was evaluated using the Kirby-Bauer method. Polymerase Chain Reaction (PCR) was employed to identify resistance and virulence genes.

**Results:**

*S. aureus* was detected in 7.5% of the samples analyzed, with higher prevalence in Clinic I and Emergency areas. The most contaminated surfaces included door handles, tables, and keyboards, identified as critical transmission points. Among the isolated strains, 66.6% were resistant to penicillin, while 100% were sensitive to methicillin and vancomycin. Virulence genes (*tst, sea*) were present in 26.6% and 13.3% of the strains, respectively. Regarding regulatory genes, *agrI* (73.3%) was the most common, followed by *agrIII*. For adhesion factors, *icaD* and *icaC* were the most frequently detected genes.

**Conclusion:**

These findings highlight the pathogenic potential of *S. aureus* and its ability to persist on inert surfaces, representing a significant risk for infection transmission.

## Introduction

*Staphylococcus aureus (S. aureus*) is the most clinically significant species within its genus, responsible for both community-acquired and hospital-acquired infections, bloodstream infections, endocarditis, pneumonia, among others (Mirzaii *et al*. 2015). *S. aureus* acts as an opportunistic pathogen, primarily affecting the immunocompromised individuals, with hospital settings being highly conducive to its spread (Garza-Velasco *et al.*, 2013).

The challenge *of S. aureus*-related hospital infections is further complicated by the rise of methicillin-resistant *S. aureus* (*MRSA*) strains, which exhibit resistance to multiple antibiotics, including macrolides, lincosamide, aminoglycosides and specially, currently available beta-lactams (Mirzaii *et al.*, 2015; Goudarzi *et al.*, 2017). Additionally, a significant increase in vancomycin-resistant S*. aureus* (*VRSA)* strains has been observed, posing a major global health threat due to their potential to increase mortality rates worldwide (Asghar *et al.*, 2023).

 Furthermore, *S. aureus* resistance is mediated by genes such as *blaZ (*penicillin resistance) (Orbe *et al.*, 2021; Ortiz *et al.*, 2023) and *mecA* (methicillin resistance). According to the World Health Organization (WHO), antimicrobial resistance is a public health threat that humanity faces (Orbe *et al*. 2021; Ortiz *et al.*, 2023), exacerbated by MRSA’s ability to form biofilms, which facilitates its survival on surfaces, resistance to multiple antibiotics, and cross-contamination, ultimately increasing morbidity and mortality rates in medical environments and even among healthy individuals ( Orbe *et al.*, 2021; Ortiz *et al.*, 2023).

In this context, health care personnel are the primary vectors of pathogen transmission, as 20-40% of nosocomial infections are transmitted through direct contact between the hands of the healthcare workers and patients or indirectly through contaminated surfaces. (Russotto *et al.*, 2015; Andrade T and Orellana B, 2019).

The pathogenicity and resistance of *S. aureus* are associated with various virulence genes. Among them, the *tst* gene is linked to the production of toxic shock syndrome toxin (TSST), responsible for toxic shock syndrome (Xie *et al.*, 2016). The *sea* gene encodes enterotoxin A, which is implicated in food poisoning and severe intestinal illnesses (Xie *et al.*, 2016). The *agr* gene regulatory system controls the expression of virulence factors (Xie *et al.*, 2016), while *ica* genes promote biofilm formation by catalyzing the production of intercellular adhesion polysaccharides (Xie *et al.*, 2016). These mechanisms facilitate adhesion, immune evasion, and toxicity in the host, increasing the risk of cross-contamination in hospital environments (Xie *et al.*, 2016).

Given that *S. aureus* represents a serious public health problem in hospital environments, this study aimed to determine its presence, antibiotic susceptibility, and the occurrence of virulence, adhesion, and regulatory genes in hospital surface samples using phenotypic and molecular techniques. Hospital surfaces in Loja, Ecuador, were specifically selected due to the lack of local data on the prevalence, resistance patterns, and genetic characteristics of this pathogen in clinical settings. This context justifies the focus of the study, as it seeks to provide relevant information to support local infection control strategies.

## Materials and Methods

### Type and design of the study

This study employed a mixed-method (qualitative-quantitative), descriptive, and non-experimental design, as no variables or experimental conditions were manipulated during the research. The study followed a cross-sectional design, as data was collected and evaluated within a defined period (Sampieri *et al.*, 2014).

### Sample collection

A total of 200 surface samples were collected from hospital environments, with 50 samples per service areas: Clinic I, Clinic II, Intensive Care Unit (ICU), and Emergency. The selected surfaces included nursing station and medical residence telephones, computer keyboards, door handles, bed railings, vital sign monitor buttons, hand sanitizer and soap dispenser, faucet handles and light switches.

Samples were collected using a swab moistened in tryptic soy broth. The collected samples were analyzed and processed at the Molecular Biology and Genetics Laboratory of Research Innovation and Technology Transfer Center at Catholic University of Cuenca.

### Isolation and Phenotypic Identification of S. aureus

The samples, collected in tryptic soy broth, were taken to the laboratory and incubated at 37°C for 24 hours. Subsequently, primary identification of the isolates was performed by evaluating colony morphology, mannitol salt agar pigmentation, gram staining and biochemical characteristics (coagulase and DNase) (Andrade *et al.*, 2023).

### Antibiotic Susceptibility

A bacterial inoculum adjusted to the 0.5 McFarland scale was used to perform the Kirby-Bauer disk diffusion method, utilizing the following antibiotic discs: cefoxitin (30 µg), penicillin (10 units), clindamycin (2 µg), erythromycin (15 µg), gentamicin (120 µg), tetracycline (30 µg), ciprofloxacin (5 µg), levofloxacin (5 µg), trimethoprim-sulfamethoxazole (25 µg), chloramphenicol (30 µg), rifampicin (5 µg) y linezolid (30 µg). The procedure followed the guidelines established by the Clinical and Laboratory Standards Institute (CLSI, 2021;Performance standards for antimicrobial susceptibility testing, 2021).

### Molecular Study

**Bacterial DNA Extraction and Molecular Identification:** The *nucA* and *femB* genes were identified following the procedures described by Andrade et al. (Andrade *et al.*, 2023).

### Genotypic Identification of Resistance Genes

The antibiotic susceptibility test results were compared with the PCR amplification of resistance genes, including *blaZ, mecA y vanA*, according to the conditions specified in **Table 1**.

**Table 1:** Primers and PCR conditions for the detection of resistance genes in *Staphylococcus aureus*.

RESISTENCE GENES AND CONTROL STRAINS	PRIMERS	AMPLICON (P.B.)	AMPLIFICATION CONDITIONS	REF.
*blaZ* ATCC 11632	F: 5´-GTTGCGAACTCTTGAATAGG-3´ R:5´GGAGAATAAGCAACTATATCATC-3´	674	94 ºC x 5 min **34 cycles of:** 94ºC x 1 min 54ºC x 1 min 72 ºC x 1min **Elongation final:** 72 ºC x 10 min	Laica *et al* (2021) (Laica *et al*., 2021)

*mecA* *ATCC 43300*	F:5´GTAGAAATGACTGAACGTCCGATGA-3´ R:5´- CCAATTCCACATTGTTTCGGTCTAA-3	310		

*vanA* *ATCC 70021*	F: 5´-GGGAAAACGACAATTGC -3´ R:5´-GTACAATGCGGCCGTTA-3	732	94 °C x 2 min **30 cycles:** 94°C x 1 min 54°C x 1 min 72°C x 1 min **Elongation final** 72°C x 10 min	Andrade *et al.,* (2023) (Andrade *et al.,* 2023)

**Identification of virulence, regulatory and adhesion genes in *S. aureus***.Using the end-point PCR technique, virulence, regulatory and adhesion genes were identified in **Table 2**.

**Table 2:** Primers and PCR conditions for the detection of virulence, regulatory and adhesion genes in Staphylococcus aureus.

TOXINS	GENES	PRIMERS 5’-3’	AMPLIFICATION CONDITIONS	AMPLICON P.B.	REF.
TSST-1	*Tst*	F: TTCACTATTTGTAAAAGTGTCAGACCCACT R: TACTAATGAATTTTTTTATCGTAAGCCCTT	**Denaturalization:** 94°C x 5 min **30 cycles of:** 94°C x 30 seg 55°C x 1 min 72°C x 1 min **Final Extension:** 72°C x 10 min	180	**Pacheco *et al* (2021)** (Pacheco *et al*., 2021) **Jarraud *et al* (2002)** (Jarraud *et al*., 2002) **Villalta *et al* (2021)** (Villalta-Calderón *et al*.. 2021)
	
PVL	*lukS-PV*	F: ATCATTAGGTAAAATGTCTGGACATGATCCA R: GCATCAASTGTATTGGATAGCAAAAGC		433	
	
Enterotoxins	*Sea*	F: TGCAGGGAACAGCTTTAGGC R: GTGTACCACCCGCACATTGA		250	**Adame et al (2021)** (Adame-Gómez et al., 2021)
	
	*seb*	F: ATTCTATTAAGGACACTAAGTTAGGG R: ATCCCGTTTCATAAGGCGAGT		400	
	
	*sec*	F: GTAAAGTTACAGGTGGCAAAACTTG R: CATATCATACCAAAAAGTATTGCCGT		297	

Accessory Regulator Gene	*agrI*	F: ATGCACATGGTGCACATGC R: GTCACAAGTACTATAAGCTGCGAT	Denaturalization: 94°C x 5 min **30 cycles of:** 94°C x 1min 57°C x 1min 72°C x 1min **Final Extension:** 72°C x 5 min	440	**Pavón *et al* (2024)** (Pavón *et al.,* 2024)
	*agr II*	F: ATGCACATGGTGCACATGC R: GTATTACTAATTGAAAAGTGCCATAGC		572	
	*agr III*	F: ATGCACATGGTGCACATGC R: CTGTTGAAAAAGTCAACTAAAAGCTC		406	

	*icaA*	F: GGTAGGTAAAGAAATTGCAAT R: AGCGTTGGGTATTCCCTCTGTCT	**Denaturalization:** 94°C x 5 min **30 cycles of:** 94 °C x 30sg 55°C x 1min 72 °C x 1,5min **Final Extension :** 72 °C x 5min	1200	**Tola *et al***. (2024) (Tola *et al*., 2024)

	*icaB*	F: AGAATCGTGAAGTATAGAAAATT R: TCTAATCTTTTTCATGGAATCCGT	**Denaturalization:** 94°C x 5 min **30 cycles of:** 94 °C x 30sg 52°C x 1min 72 °C x 1,5min **Final Extension:** 72 °C x 5min	900	

	*icaC*	F: ATGGGACGGATTCCATGAAAAAGA R: TAATAAGCATTAATGTTCAATT	**Denaturalization:** 94°C x 5 min **30 cycles of:** 94 °C x 30sg 50°C x 1min 72 °C x 1,5min **Final Extension:** 72 °C x 5min	1100	

	*icaD*	F: ATGGTCAAGCCCAGACAGAG R: CGTGTTTTCAACATTTAATGCAA	**Denaturalization:** 94°C x 5min 50 cycles of: 94 °C x 30seg 55,5 °C x 30seg 72 °C x 30seg **Final Extension:** 72 °C x 1min	198	

## Results

The frequency of *S. aureus* isolated from hospital surfaces was 7.5% (15/200), confirmed through microbiological methods (coagulase and DNase) and molecular methods *(nucA* and *femB* genes).

The results show that the areas with the highest frequency of *S. aureus* were Clinic I and Emergency. **Table 3**.

**Table 3:** Distribution of *S. aureus* in surface samples from a hospital center.

SURFACE	N^O^. OF ISOLATED SAMPLES	AREA	FREQUENCY
Bed railing 403D	1	Clinic I	10% (5/50)

Door handle 405	1		

Food table 405A	1		

Infusion pump buttons 406D	1		

Door handle 406	1		

Chair armrest 303	1	Clinic II	6% (3/50)

Light switch 303	1		

Medical residence: Table	1		

Cubicle I: Door handle	1	ICU	4% (2/50)

Medical residence: Keyboard 1	1		

Sample collection: Chair armrest 2	1	Emergency	10% (5/50)

Nursing station: Keyboard 1	1		

Nursing station: table	1		

Scale 2: Weight beam	1		

Medical supplies: Keyboard	1		

### Antibiotic Susceptibility

The disk diffusion susceptibility test indicated that, among hospital surface samples, 10 out of 15 (66.67%) *S. aureus* positive strains were resistant to penicillin. Only one strain showed a positive D-test (Clindamycin resistance induced by erythromycin). Additionally, one strain exhibited intermediate resistance to rifampin, levofloxacin and chloramphenicol. All strains (100%) were sensitive to cefoxitin, gentamicin, tetracycline, ciprofloxacin, trimethoprim-sulfamethoxazole and linezolid as detailed in **Table 4**.

**Table 4:** Antibiotic susceptibility of *S. aureus* strains presents on hospital surfaces.

Surface	P	FOX	D-test	CN	TE	CIP	LEV	SXT	C	RD	LIZD
Bed railing 403D	S	S	+	S	S	S	S	S	S	S	S

Door handle 405	R	S	-	S	S	S	S	S	S	S	S

Food table 405A	R	S	-	S	S	S	S	S	S	S	S

Infusion pump buttons 406D	R	S	-	S	S	S	S	S	S	S	S

Door handle 406	R	S	-	S	S	S	S	S	S	S	S

Chair armrest 303	R	S	-	S	S	S	S	S	S	S	S

Light switch 303	S	S	-	S	S	S	S	S	S	S	S

Medical residence: Table	R	S	-	S	S	S	S	S	S	S	S

Cubicle I: Door handle	R	S	-	S	S	S	S	S	S	S	S

Medical residence: Keyboard 1	R	S	-	S	S	S	S	S	S	S	S

Sample collection: Chair armrest 2	R	S	-	S	S	S	S	S	S	S	S

Nursing station: Keyboard 1	R	S	-	S	S	S	S	S	S	S	S

Nursing station: table	S	S	-	S	S	S	S	S	S	S	S

Scale 2: Weight beam	S	S	-	S	S	S	S	S	S	S	S

Medical supplies: Keyboard	S	S	-	S	S	S	I	S	I	I	R

**P:** Penicillin, **FOX:** Cefoxitin, **DA:** Clindamycin, **E:** Erythromycin, **CN:** Gentamicin, **TE:** Tetracycline, **CIP:** Ciprofloxacin, **LEV:** Levofloxacin, **SXT:** Trimethoprim sulfamethoxazole, **C:** Chloramphenicol, **RD:**Rifampicin, **LIZD:** Linezolid. **R:** Resistant, **S:** Sensitive, **I:** Intermedium, Negative: (-), Positive: (+).

**Table 5:** Virulence, regulatory and adhesion genes detected in each G *S. aureus* strain

SAMPLE ORIGIN	AREA	ENTEROTOXINS	ACCESSORY REGULATORY GENE	ADHESION FACTORS
**Bed railing**	Clinic I	*tst, sea,*	*agr III*	*icaB, icaC, icaD*
	
**Door handle**		*-*	*agrI*	*icaA, icaB, icaC, icaD*
	
**Food table**		*-*	*agrI*	*icaA, icaB, icaC, IcaD*
	
**Infusion pump buttons**		*-*	*agrI*	*icaA, icaB, icaD*
	
**Door handle**		*-*	*agrI*	*icaB, icaC, icaD*

**Chair armrest**	Clinic II	*-*	*-*	*icaD*
	
**Light switch**		*-*	*-*	*icaD*
	
**Medical residence: Table**		*Tst*	*agrI*	*icaA, icaB, icaC, icaD*

**Cubicle 1: Door handle**	UCI	*Tst*	*agrI*	*icaA, icaB, icaC, icaD*
	
**Medical residence: keyboard 1**		*tst, sea*	*agr III*	*icaA, icaB, icaC, icaD*

**Sample collection: Chair armrest 2**	Emergency	*-*	*agrI*	*icaA, icaB, icaC, icaD*
	
**Nursing station: Keyboard 1**		*-*	*agrI*	*icaA, icaB, icaC, icaD*
	
**Nursing station: table**		*-*	*agrI*	*icaA, icaB, icaC, icaD*
	
**Scale 2: Weight beam**		*-*	*agrI*	*icaA, icaC, icaD*
	
**Medical supplies: Keyboard**		*-*	*agrI*	*icaA, icaB, icaC, icaD*

The detection of resistance genes was consistent with the phenotypically identified penicillin-resistant strains as they amplified for the *blaZ* gene (See Figure 2).

**Figure 1:**
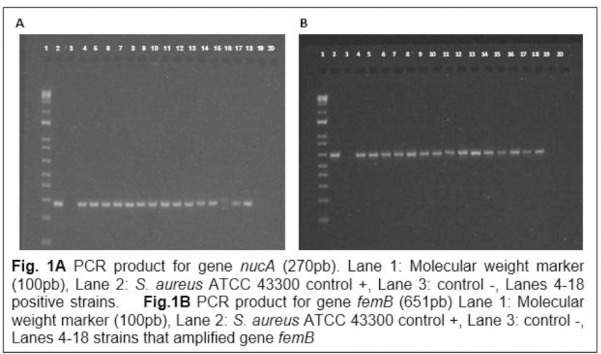
1.1% agarose gel electrophoresis of the *nucA* and *femB* genes.

**Figure 2:**
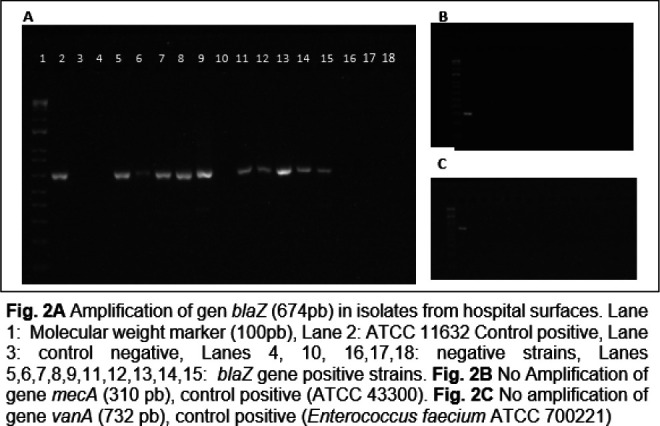
1% agarose gel electrophoresis of the *blaZ, mecA* and *vanA* genes

Resistance to methicillin and vancomycin was also analyzed through the identification of the *mecA* and *vanA* genes respectively; however, none of the strains amplified for these genes.

### Identification of virulence, regulatory and adhesion genes

The virulence gene detection assay showed that 4/15 isolates (26.6%) carried the *tst* gene. The *sea* gene was present in 2/15 isolates (13.3%). In relation to the amplification of accessory regulatory genes (*agr)*, 11/15 isolates (73.3%) amplified for the *agrI* gene, only 2/15 (13.3%) amplified for the *agrIII* gene, while *agrII* did not amplify in any isolate.

Regarding adhesion factors, *icaA* and *icaB* genes were detected in 111/15 (73.3%) isolates. The *icaC* gene was detected in 12/15 (80%). Finally, the *icaD* gene was found in 100% of the isolates.

The *eta*, *etb*, *seb*, *sec* and *lukS-PV* genes were not detected in any of the 15 analyzed isolates.

## Discussion

In the present study**,** *S. aureus* colonies were isolated, their antibiotic susceptibility and virulence factors were determined from hospital surfaces to confirm their relevance at a nosocomial level (Partida *et al.*, 2015).

Literature indicates that hospital personnel can act as carriers and vectors for the dissemination of *S. aureus* through their hands and clothing, aligning with our results, which showed the presence of microorganism on surfaces with high manual contact (Shidiki *et al.*, 2019). This finding is particularly concerning, as the surfaces serve as potential transmission, hotspots, highlighting the urgent need to implement strict hygiene and sanitation protocols, as well as conducting regular cultures to identify carriers among healthcare staff. Similarly, the detection of *S. aureus* in critical areas, such as the ICU, although less frequent, remains a threat due to a vulnerability of patient in this zone (Garza-Velasco *et al.*, 2013).

Previous studies report variations in the frequency of *S. aureus* on hospital surfaces. In a hospital in Cali, its presence was 12.2% (20/167), with a significant concentration in the ICU (Cháves-Visas *et al.*, 2017). In London, French *et al*. in 2004 found a significantly higher concentration rate of 27% (French *et al.*, 2004), whereas the study reported a lower value of 7.5% (15/200). Similar results were found in Dublin in 2021, with 7.7% (62/810) in high contact sites (Kinnevey *et al.*, 2021). However, studies conducted in Cuenca, Ecuador and the Netherlands reported lower values, with 3% (Orbe *et al.*, 2021) and 2,8% (Schoor *et al.*, 2023) respectively, with a higher frequency in the emergency area. The differences among these results may be due to multiple factors, such as environmental conditions, cleaning strategies, and disinfection protocols implemented in each hospital.

Regarding antibiotic susceptibility, genotypic identification via PCR confirmed the presence of the *blaZ* (penicillin resistance) gene and the absence of the *mecA* (methicillin resistance) and *vanA* (vancomycin resistance) genes in the isolated strains, confirming the phenotypic results these genes are well known for their role in *S. aureus* resistance mechanisms against different antibiotics (Orbe *et al.*, 2021; Ortiz *et al.*, 2023).

Our results indicate that 66.7% of isolates were resistant to penicillin, consistent with the literature, which highlights a significant prevalence of *S. aureus* strains producing β-lactamase (Ortiz *et al.*, 2023). In Azuay, Ecuador, Sari *et al*. (2023) reported 62.5% penicillin resistance and 100% susceptibility to cephalexin, clindamycin, and erythromycin (Cháves-Visas *et al.*, 2017), aligning with our findings.

Inducible clindamycin resistance (iMLSB) is significant because misidentification can lead to treatment failures with this antibiotic (Schoor *et al.*, 2023). The present study showed a low frequency of iMLSB (6.6%), compared to previous studies, such as one conducted in a tertiary hospital in Kathmandu, Nepal (2021), where 36.5% of *S. aureus* exhibited this phenotype (Thapa *et al.*, 2021). Similarly, in clinical samples from the National Medical College and the Teaching Hospital of Nepal (2019), 23.22% of *S. aureus* isolates were iMLSB-positive, with a higher percentage in MRSA strains than in MSSA strains (Shidiki *et al.*, 2019).

The intermediate resistance observed in one string to levofloxacin, chloramphenicol, and rifampin, highlights the need for continuous surveillance, and prudent use of these antibiotics. On the other hand, 100% of these isolates were sensitive to cefoxitin, gentamicin, tetracycline, ciprofloxacin, trimethoprim-sulfamethoxazole, and linezolid, indicating that these antibiotics still maintain significant effectiveness against *S. aureus*. These results are similar to those of a study conducted in Romania (2023), which found a 10% resistance rate in *S. aureus* strains against ciprofloxacin, moxifloxacin, gentamicin, rifampin and trimethoprim-sulfamethoxazole (Tălăpan *et al.*, 2023).

In our study, we also evaluated virulence genes such as *tst, sea* and *ica*, which are associated with S. aureus’s ability to cause severe infections and evade the immune system (Jarraud *et al.*, 2002; Rasmi *et al.*, 2022). Their presence indicates the pathogenic potential of the isolates and their capacity to cause severe infections in hospitalized patients.

The genotypic study of *S. aureus* found the *tst* gene in 26.6% of isolates, lower percentage compared to 50% reported in a hospital in Azuay in 2021 (Villalta-Calderón *et al.*, 2021). This suggests that not all *S. aureus* isolates from hospitals surfaces have the same ability to cause toxic shock syndrome (Xie *et al.*, 2016).

The *sea* gene was detected in 13.3% of isolates. In contrast to our results, Xie *et al*. (2016) in China reported a high prevalence of the sea gene (59.7%) in clinical isolates from patients with surgical wound infections (Xie *et al.*, 2016). Similarly, Ramsi *et al*. (2022) in a hospital in Mina, Egypt, found a 72.9% prevalence of this gene in clinical samples (Rasmi *et al.*, 2022). These differences may be due to the origin of the samples, as the isolates in these studies were MRSA from clinical samples, which could explain the higher prevalence of *sea*.

In this study, a significant prevalence of the *agrI* gene (73.3%) was observed in the isolates, suggesting that this regulator may play an important role in controlling virulence factor expression in these strains. However, the low presence of *agrIII* (13.3) and the absence of *agrII* suggests variations in the virulence regulation mechanism among different *S. aureus* strains. A similar study in Cali reported the presence of *agrI, agrII* and *agrIII* in 51.4%, 28.6% and 25.7%, respectively with a higher prevalence in MSSA strains (Cháves-Visas *et al.*, 2017).

In our study, the high prevalence of *icaA* and *icaB* genes (73.3%) and *icaD* (100%) suggests a considerable capacity for biofilm information on hospital surfaces. Additionally, the *icaC* gene was present in 80% of isolates, reinforcing this idea due to its role in the synthesis of polysaccharides that facilitate bacterial adhesion.

Studies conducted on clinical samples in Quito and Puyo (Ecuador) reported lower percentages compares to ours with *icaA, ica, icaC* and *icaD* detected in 70%, 35%, 26% and 66%, respectively (Sanguano *et al.*, 2021). These differences may be due to the origin of the isolates, as in our case, they were obtained from surfaces, whereas the mentioned study analyzed clinical samples.

## Conclusion

The present study evidenced the presence of *S. aureus* on surfaces from various hospital services sampled and its ability to survive in inanimate environments, suggesting that it represents a significant risk factor for the development of hospital-acquired infections, especially in immunosuppressed patients. The high prevalence of penicillin-resistant strains indicates that this drug should not be considered for the treatment of *S. aureus* infections. Similarly, the presence of different virulence factors is associated with pathogenic potential, adhesion capacity, biofilm formation and resistance to certain antibiotics. The constant movement of hospital healthcare personnel highlights the need for strategies to prevent and control the dissemination of *S. aureus*.

### Ethical responsibilities

**Protection of people and animals:** The authors declare that no experiments were conducted on humans or animals during this study.

**Protection of vulnerable population**: Not applicable

**Confidentiality and privacy:** The authors declare that they have followed the established protocols related to information protection and data disclosure.

**Funding:** The authors acknowledge the use of funds provided by the Universidad Católica de Cuenca for the research project titled “Evaluación de la contaminación bacteriana (*Staphylococcus aureus* y *Escherichia coli*) en superficies inertes de hospitales y análisis de la resistencia a antibióticos”, PIC5P23-63, which supported the development of this study.

### Conflict of Interests

The authors declare that there is no conflicts of interest associated with this study.

### Author Contribution

Conceptualization: C.L., J.O.; Methodology: C.L., C.A., J.O., P.O.; K.T.; Recourses: C.L., C.A., PO.; Supervision: C.A; Validation: C.A.; Investigation: C.L., C.A. All authors contributed, read and approved the submitted version of the manuscript.

**List of Abbreviations:**
agr: Accessory Gene Regulator;
ATCC: American Type Culture Collection;
blaZ: Beta-lactamase gene;
C: Chloramphenicol;
CIITT: Research, Innovation and Technology Transfer Center;
CLSI: Clinical and Laboratory Standards Institute;
CN: Gentamicin;
CIP: Ciprofloxacin;
DA: Clindamycin;
DNase: Deoxyribonuclease;
FOX: Cefoxitin;
ICA: Intercellular Adhesion (*icaA, icaB, icaC, icaD* genes)
ICU: Intensive Care Unit;
iMLSB: Inducible Macrolide-Lincosamide-Streptogramin B resistance;
LIZD: Linezolid;
LEV: Levofloxacin;
MRSA: Methicillin-resistant *Staphylococcus aureus;*
MSSA: Methicillin-susceptible *Staphylococcus aureus;*
nucA: Thermonuclease gene specific to *S. aureus;*
P: Penicillin;
PCR: Polymerase Chain Reaction;
RD: Rifampicin;
S. aureus: *Staphylococcus aureus;*
SXT: Trimethoprim-sulfamethoxazole;
tst: Toxic Shock Syndrome Toxin-1 gene;
TE: Tetracycline;
TSST: Toxic Shock Syndrome Toxin;
vanA: Vancomycin resistance gene;
VRSA: Vancomycin-resistant *Staphylococcus aureus*
